# LRRK2 Deficiency Aggravates Sleep Deprivation-Induced Cognitive Loss by Perturbing Synaptic Pruning in Mice

**DOI:** 10.3390/brainsci12091200

**Published:** 2022-09-06

**Authors:** Xiaojuan Cheng, Xilin Wu, Yuying Zhang, Weian Li, Linjuan Feng, Hanlin You, Siyu Yang, Dongping Yang, Xiaochun Chen, Xiaodong Pan

**Affiliations:** 1 Department of Neurology, Center for Cognitive Neurology, Institute of Clinical Neurology, Fujian Medical University Union Hospital, Fuzhou 350001, China; 2 Fujian Institute of Geriatrics, Fujian Medical University Union Hospital, Fuzhou 350001, China; 3 Fujian Key Laboratory of Molecular Neurology, Fujian Medical University, Fuzhou 350001, China; 4 Key Laboratory of Brain Aging and Neurodegenerative Diseases, Fujian Medical University, Fuzhou 350001, China; 5 School of Advanced Manufacturing, Fuzhou University, Fuzhou 350108, China; 6 Research Institute of Artificial Intelligence, Zhejiang Laboratory, Hangzhou 310027, China

**Keywords:** LRRK2 deficiency, sleep deprivation, REM/NREM, microglial synaptic pruning

## Abstract

Mutations of the *leucine-rich repeat kinase 2* (LRRK2) gene are associated with pronounced sleep disorders or cognitive dysfunction in neurodegenerative diseases. However, the effects of LRRK2 deficiency on sleep rhythms and sleep deprivation-related cognitive changes, and the relevant underlying mechanism, remain unrevealed. In this study, *Lrrk2^-/-^* and *Lrrk2^+/+^* mice were subjected to normal sleep (S) or sleep deprivation (SD). Sleep recording, behavioral testing, Golgi-cox staining, immunofluorescence, and real-time PCR were employed to evaluate the impacts of LRRK2 deficiency on sleep behaviors and to investigate the underlying mechanisms. The results showed that after SD, LRRK2-deficient mice displayed lengthened NREM and shortened REM, and reported decreased dendritic spines, increased microglial activation, and synaptic endocytosis in the prefrontal cortex. Meanwhile, after SD, LRRK2 deficiency aggravated cognitive impairments, especially in the recall memory cued by fear conditioning test. Our findings evidence that LRRK2 modulates REM/NREM sleep and its deficiency may exacerbate sleep deprivation-related cognitive disorders by perturbing synaptic plasticity and microglial synaptic pruning in mice.

## 1. Introduction

Sleep disorders and cognitive dysfunction are characteristic symptoms of neurodegenerative disorders, such as dementia with Lewy bodies (DLB) and Parkinson’s disease (PD). Available literature indicates that the primary culprit for monogenic PD is the mutations in *leucine-rich repeat kinase 2* (LRRK2) genes [1,2,3]. PD carriers of these mutations often complain of insomnia, which increases their risk for dementia [4,5]. Evidence suggests that isolated rapid-eye-movement sleep behavior disorder (iRBD) may serve as a hallmark for prodromal synucleinopathy [6], while a decreased RBD risk is closely connected with the LRRK2 protective haplotype [7]. Sleep regulation has a strong genetic component [8]. However, it remains blurred with regards to the effects of LRRK2 deficiency on sleep disorders and cognition loss, and the underlying mechanisms.

Given the electromyogram (EMG) and electroencephalogram (EEG) recordings, sleep is broadly categorized into two phases: rapid eye movement (REM) and nonrapid eye movement (NREM) sleep [9]. During NREM sleep, decreased EEG activity, mainly with lower delta frequency in the frontal lobe region [10], indicates lower physiological function, which is indicative of mild cognitive impairment in patients with Parkinsonism [11]. Moreover, REM sleep has been documented to consolidate spatial and emotional memory [12]. As an important component of the default mode network, the prefrontal cortex (PFC) is a vital structural basis for sleep and cognition. Accumulating evidence suggests that sleep is essential for the proper functioning of cognition, including learning and memory, and modulates synaptic plasticity and spine homeostasis. Bidirectionally, sleep deprivation alters structural plasticity, impairs the removal of neural debris from the affected axons, and induces age-dependent microglial reactions [13,14,15]. Heavily enriched in microglia, especially in those of the PFC, LRRK2 can disrupt synaptic activity within the complex neural circuitry [16]. However, little is available to elucidate the role of *Lrrk2* in REM/NREM sleep.

In the present study, a LRRK2 knockout mouse model was subjected to sleep deprivation to investigate the effects of LRRK2 deficiency on REM/NREM sleep and sleep deprivation-induced cognitive dysfunction, and the related underlying mechanism. The findings of this investigation may provide potential therapeutic implications for LRRK2 as a target for sleep deprivation-mediated cognitive loss in patients with neurodegenerative disorders.

## 2. Materials and Methods

### 2.1. Transgenic Mice and Ethics Approval

Thirteen-month-old *Lrrk2* knockout mice (*Lrrk2^-/-^*, C57BL/6-*Lrrk2^tm1.1Mjff^*/J; N = 64) (stock No. 012453, *Lrrk2^-/-^*) and age-matched wild-type (*Lrrk2^+/+^*; N = 67) were raised in recording boxes during the experiment (with the light on at 8:00 A.M. on a 12 light/dark cycle and the temperature set at 23 ± 1 °C). The animals were allowed access to water and food ad libitum. Male and female transgenic (*Lrrk2^-/-^*) and wild-type (*Lrrk2^+/+^*) littermate mice (1:1 ratio for sex balance) were bred from deficit-free heterozygotes. The animal experimental procedures followed the guidelines of the Institutional Animal Care and Use Committee of Fujian Medical University (IACUC number: FJMU IACUC 2021-0314).

### 2.2. Experimental Design

Sleep deprivation (SD) experiments were conducted on a treadmill (SANS SA101; Saiangsi, Inc., Nanjing, China), with the activity wheel generating a slow rotation (~2.5 m/min). All SD mice were deprived of sleep for 18 h/day (from 6:00 a.m. to 12:00 p.m.) for 3 days. Mice undergoing normal sleep (S group) were raised in the same environment and allowed a normal sleep phase. Food and water were freely available for each group. All behavioral tests were performed between 9 a.m. and 2 p.m.

### 2.3. Surgery Implantation

A ketamine (100 mg kg^−1^)/xylazine (20 mg kg^−1^) cocktail was administered to anesthetize the mice. EEG and EMG electrodes were implanted into the anesthetized *Lrrk2^+/+^* and *Lrrk2^-/-^* mice. One electrode was placed 1.0 mm lateral to the central suture and 1.5 mm anterior to the bregma (on the PFC). Another two electrodes were located at 1.5 mm on either side of the central suture and 4.0 mm posterior to the bregma. Three stainless steel screws (M1; Tianjin, China) were implanted into the skull (one EEG screw was placed on the PFC, one used as a reference, and one as a ground). The EMG activity was monitored using a silver wire (Cat No. 786000; A-M Systems, Sequim, WA, USA) that was inserted bilaterally into the nuchal muscle. Mice were allowed to recuperate for at least 10 days prior to the recordings of their sleep activities.

### 2.4. EEG/EMG Recordings

**Figure 1:**
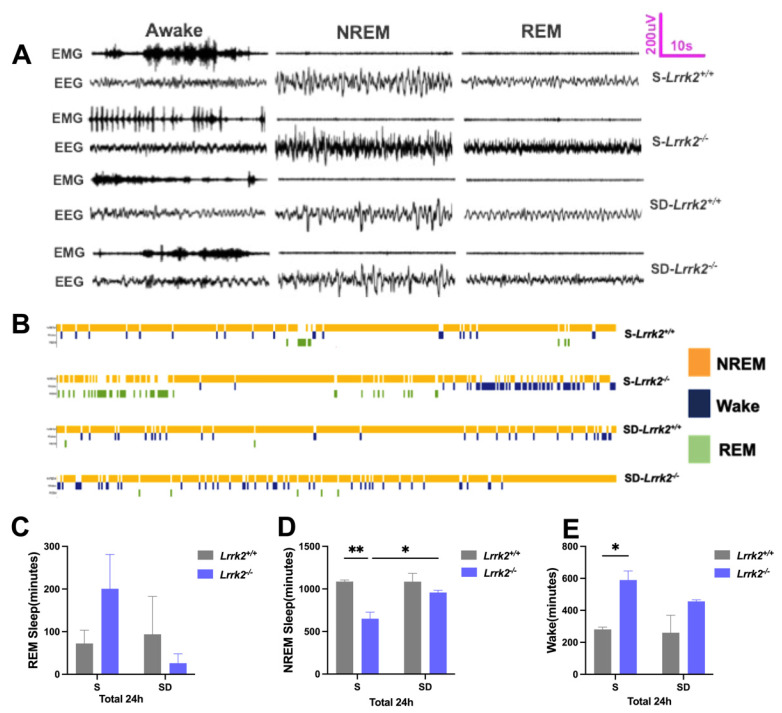
The inverted effects of LRRK2 deficiency on the NREM sleep before and after SD. (**A**) Representative EEG and EMG traces recorded from the S-*Lrrk2^+/+^*, S-*Lrrk2^-/-^*, SD-*Lrrk2^+/+^*, and SD-*Lrrk2^-/-^* groups during the REM sleep, NREM sleep, and wakefulness. (**B**) Channel assignments corresponding to color coding (wakefulness, blue; NREM sleep, yellow; REM sleep, green). Individual hypnograms for S-*Lrrk2^+/+^*, S-*Lrrk2^-/-^*, SD-*Lrrk2^+/+^* and SD-*Lrrk2^-/-^* groups under undisturbed conditions. (**C**–**E**) The duration of REM sleep and NREM sleep over the 24 h recording period in S-*Lrrk2^+/+^*, S-*Lrrk2^-/-^*, SD-*Lrrk2^+/+^*, and SD-*Lrrk2^-/-^* groups. N = 3–4 mice per group. Data are plotted as mean ± SEM and were analyzed by *two-way* ANOVA with Tukey’s multiple comparisons test (**C**–**E**), * *p* < 0.05, ** *p* < 0.01.

Ten days after the surgery, data acquisition was performed for all *Lrrk2^+/+^* and *Lrrk2^-/-^* mice (including the normal sleep (S) and sleep deprivation (SD) groups) with the Lab Chart Acquisition software (Pinnacle Technologies, Norwood, MA, USA). The recordings were subsequently amplified (ML4818; AD Instruments, Colorado Springs, CO, USA); EEG signals were filtered at 0.5 Hz–100 Hz and EMG signals at 10 Hz–1K Hz. Both channels were collected continuously for 24 h. After data collection, all waveforms were sorted by an experienced analyst [17,18]. The epochs were classified as NREM sleep, characteristic of large, slow EEG waves (<4 Hz) and only a weak or no EMG signal, and as REM sleep, typical of rapid low-voltage EEG waves (6–10 Hz) and low EMG amplitude. Otherwise, they were classified as wakefulness (Figure 1A,B).

### 2.5. Fear Conditioning

The procedures of the fear conditioning were performed as described previously [19]. The S group followed the circadian rhythm and completed the test during the day, while the SD group accomplished the test after the 54-h sleep deprivation and an 8 h sleep recovery.

### 2.6. Open-Field Test

As previously described [19], the anxiety and activity levels of mice were examined with the Flex-Field activity system (SANS SA215; Saiangsi, Inc., China). The animals were placed in a square arena (50 × 50 × 50 cm^3^) of opaque black plexiglass with a central area of 18 × 18 cm^2^. The 10 min movement of the mouse in the unit was traced and quantified with the relevant software.

### 2.7. Tissue Collection and Processing

Mice were euthanized with a ketamine (100 mg kg ^−1^)/xylazine (20 mg kg ^−1^) cocktail and transcardially perfused with sterile PBS. Brains were removed, divided, snap frozen, and stored at −80 °C for further analysis. The tissues used for immunohistochemistry, Golgi staining, and molecular study were obtained from the animals undergoing the behavior testing.

### 2.8. RNA Extraction and Real-Time PCR

The procedures of RNA extraction, cDNA production, and SYBR Green-based qPCR were performed as described previously [20]. Each sample was normalized to the mRNA levels of house-keeping gene GAPDH. Primers used were as follows:

1. IL-1 (sense 5-ATCAGCAACGTCAAGCAACG-3 and anti-sense 5-GGTTGGATGGTCTCTTCCAGA-3),
2. IL-6 (sense 5-TAGTCCTTCCTACCCCAATTTCC-3 and anti-sense 5-TTGGTCCTTAGCCACTCCTTC-3),
3. IL-4 (sense 5-GCAACGAAGAACACCACAGA-3 and anti-sense 5-TGCAGCTCCATGAGAACACT-3),
4. GAPDH (sense 5-CAGTGGCAAAGTGGAGATTGTTG-3 and anti-sense 5-CTCGCTCCTGGAAGATGGTGAT-3).

### 2.9. Immunohistological Analysis

Tissues were postfixed in 4% PFA for 6 h, washed, transferred to 30% sucrose solution, and subsequently sectioned at 40 μm with a freezing microtome (Leica Biosystems). Sections (6 sections per animal) were incubated in primary antibodies at 4 °C overnight and subsequently in corresponding secondary antibodies. In case of need, counter-staining was performed with DAPI. The images were obtained by the confocal microscopy (LSM 780, Zeiss). Scans of Z-stacks of 20~25 slices were attained at intervals (0.8~1.0 µm) and images of the whole section were processed with maximum-intensity projections. Serial coronal sections were acquired from bregma 2.8 to 1.54 mm.

The antibody dilutions used were as follows: Iba1 (#019-19741, rabbit, 1:1000; Wako Chemicals; ab5076, goat, 1:300; Abcam), CD68 (MCA1957T, Rat, 1:500; Bio-raid), Vglut1 (135304, Guinea pigs, 1:2000; Synaptic System), PSD95 (RL238256, rabbit, 1:500; Thermo Fisher).

As described previously [20,21], the 20–25 images of the Z-stack (0.8 μm thick each) were analyzed on maximum-intensity projections (Z-project, Maximum Intensity function in FIJI). The images of Iba1-1^+^, CD68^+^, PSD95^+^, Vglut1^+^ were captured using a 63 × oil objective and 3D reconstructed and analyzed using the FIJI and Imaris software (7.4.2).

### 2.10. Dendritic Spine Analysis

Golgi staining was performed following the manufacturer’s instructions (Hito Golgi-Cox OptimStain^TM^ Kit, Hitobiotec, Kingsport, TN, USA). Golgi-stained sections were imaged under bright-field illumination (T-PMT) on a Zeiss microscope system (LSM 780) with a 100 ×/1.4 oil immersion lens. Each image consisted of a stack of scans taken across the z-plane of the PFC. The spine density was analyzed with the FIJI software (Fiji, ImageJ 1.46, NIH, Bethesda, MD, USA).

### 2.11. Statistical Analysis

Prism GraphPad 8.0 (San Diego, CA, USA) was employed for the statistical analyses. The means of different groups were compared and analyzed by *two-way* ANOVA and post hoc analysis. In all figures, data were represented as means ±SEM. A statistical significance was set at *p* < 0.05.

## 3. Results

### 3.1. LRRK2 Deficiency Produces Inverted Effects on the NREM Sleep before and after SD

We evaluated whether LRRK2 deficiency affects REM/NREM sleep. The changes in REM and NREM sleep were monitored (Figure 1A,B). Compared with that of the S-*Lrrk2*^-/-^ group, REM sleep in the SD-*Lrrk2*^-/-^ group decreased, though without no significance (Figure 1C). The results demonstrated that, compared with the S-*Lrrk2^+/+^* group, the S-*Lrrk2*^-/-^ group showed decreased NREM sleep (*p* = 0.0058) (Figure 1D); compared with the S-*Lrrk2*^-/-^ group, the SD-*Lrrk2*^-/-^ group reported significantly increased NREM sleep (*p* = 0.0383) (Figure 1D); and compared with the S-*Lrrk2*^+/+^ group, the S-*Lrrk2*^-/-^ group displayed apparently increased wakefulness (*p* = 0.0329) (Figure 1E). These data suggest that LRRK2 deficiency contributes to a decrease in NREM sleep; however, during SD-induced stress, LRRK2 deficiency impacts the regulation of NREM sleep.

### 3.2. LRRK2 Deficiency Deceases Dendritic Spine Density after SD

**Figure 2:**
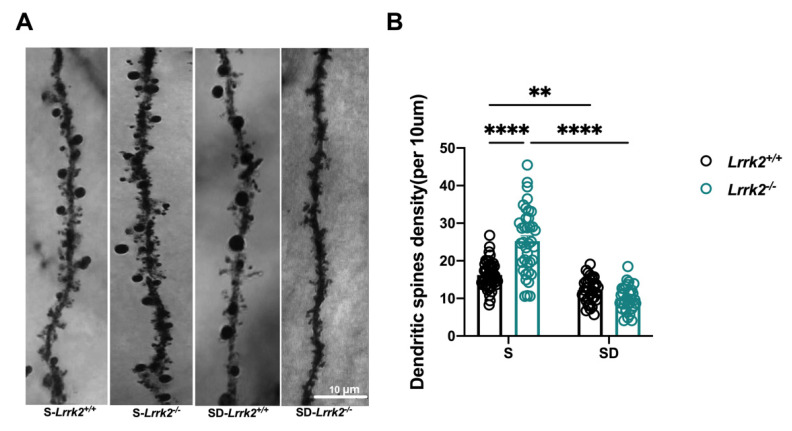
The LRRK2 deficiency-induced decease in dendritic spinal density after SD. (**A**) Representative images of secondary branches of apical dendrites by Golgi-staining, original magnification 100 × Oil; Scale bar = 10 μm. (**B**) Relative quantification of dendritic spines in the PFC: N = 4 mice, n = 34~48 dendrites. Data are plotted as mean ± SEM. ** *p* < 0.01, **** *p* < 0.0001, by two-way ANOVA analysis with Tukey’s multiple comparisons test.

Considering that sleep is equilibrated by regulating synaptic plasticity and dendritic spine [22] and SD may affect the dendritic spines [13], we explored whether LRRK2 deficiency coupled by SD affects the dendritic structure formation in the PFC. Golgi staining was implemented to calculate the number of dendritic spines in the PFC following SD (Figure 2A). Compared with the S-*Lrrk2*^+/+^ mice, the SD-*Lrrk2*^+/+^ mice displayed a noticeable decline in spine density (*p* = 0.0041) (Figure 2B). Of note, similar differences were observed in the LRRK2 deficiency groups, with a significantly lower spinal density in the SD-*Lrrk2*^-/-^ mice than in the S-*Lrrk2*^-/-^ mice (*p* < 0.0001) (Figure 2B). Moreover, the spinal density significantly increased in the S-*Lrrk2*^-/-^ mice when compared with that of the S-*Lrrk2*^+/+^ mice (*p* < 0.0001) (Figure 2B).

### 3.3. LRRK2 Deficiency Aggravates Microglial Activation after SD

**Figure 3:**
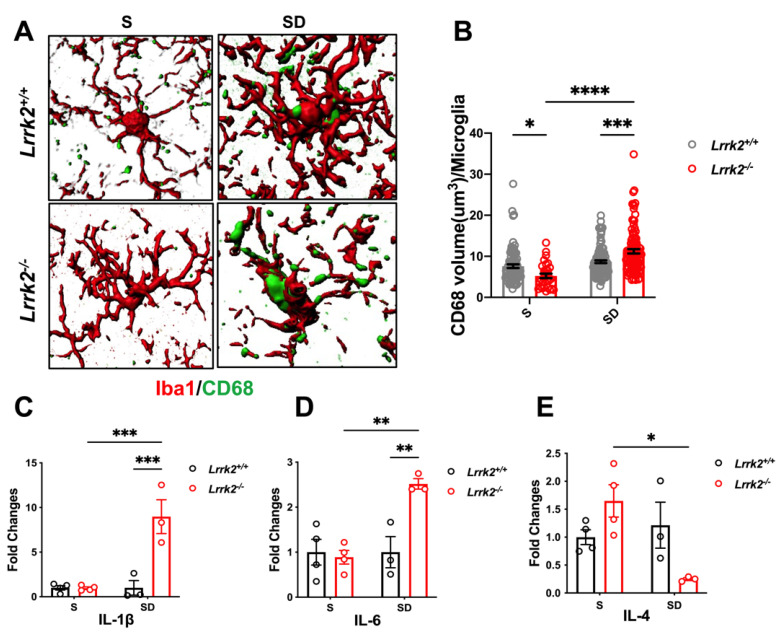
The aggravated microglial activation by LRRK2 deficiency after SD. (**A**) Representative 3-dimensional reconstructions indicating CD68^+^ (green) structures within Iba1^+^ (red) microglia in the PFC. (**B**) The quantified total volume of CD68^+^ structures per microglia cell. S-*Lrrk2^+/+^* group, N = 3 mice, n = 88 cells; SD-*Lrrk2^+/+^* group, N = 5 mice, n = 115 cells; S-*Lrrk2^-^*^/-^ group, N = 3 mice, n = 31 cells; SD-*Lrrk2^-^*^/-^ group, N = 5 mice, n = 104 cells. Data are presented as mean ± SEM. * *p* < 0.05, *** *p* < 0.001, **** *p* < 0.0001, by *two-way* ANOVA with Tukey’s multiple comparisons test. (**C**–**E**) Relative mRNA expressions of IL-4, IL-6, and IL-1β from the PFC of the S-*Lrrk2^+/+^*, SD-*Lrrk2^+/+^*, S-*Lrrk2^-/^*^-^, SD-*Lrrk2^-/-^* mice, N = 3 to 4 mice per group. Data are presented as mean ± SEM. ** *p* < 0.01, *** *p* < 0.001, by *two-way* ANOVA with Tukey’s multiple comparisons test.

Subsequently, efforts were made to explain the profound synaptic damage observed in the LRRK2 deficiency mice after SD. As studies have documented microglial involvement in spinal shrinkage and elimination [23,24], we next explored whether LRRK2 deficiency may result in the transition of microglial states in both S and SD. We evaluated the expression of the phagocytic marker CD68 in the microglia (Figure 3A). Compared with that of the S-*Lrrk2*^+/+^ counterparts, CD68 was apparently decreased in the S-*Lrrk2*^-/-^ mice (*p* = 0.0473) (Figure 3B). Surprisingly, compared with both the SD-*Lrrk2*^+/+^ group and the S-*Lrrk2*^-/-^ mice, the SD-*Lrrk2*^-/-^ group reported a significant increase in CD68 expression in the microglia (*p* = 0.0001 and *p* < 0.0001, respectively) (Figure 3B). The above results suggest that after SD, LRRK2 deficiency seems to trigger a greater immune response, which may be an important driving force in SD-associated pathophysiology. To verify this possibility, we quantified the mRNA expressions of interleukin 4 (IL-4), interleukin 6 (IL-6), and interleukin 1β (IL-1β). In the PFC, the expressions of IL-1β (*p* = 0.0004) (Figure 3C) and IL-6 (*p* = 0.0039) (Figure 3D) were significantly higher in the SD-*Lrrk2*^-/-^ mice than in the S-*Lrrk2*^-/-^ counterparts. Compared with that of the S- *Lrrk2*^-/-^ group, IL-4 decreased in the SD-*Lrrk2*^-/-^ mice (*p* = 0.0048) (Figure 3E). Altogether, these results evidence that after SD, LRRK2 deficiency may induce and exacerbate malignant neuroinflammation, particularly in the PFC.

### 3.4. LRRK2 Deficiency Increases Synaptic Pruning by Microglia after SD

**Figure 4:**
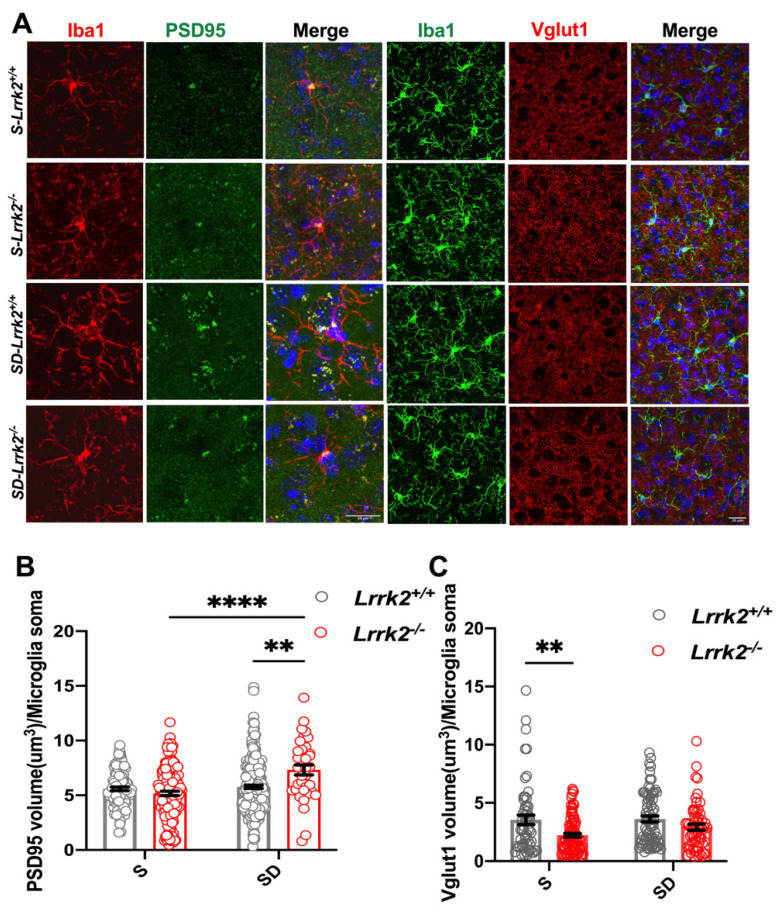
The increased microglial synaptic pruning by LRRK2 deficiency after SD. (**A**) Representative image of PSD 95 or Vglut1 merged with microglia (Iba1^+^) in the PFC sections from S-*Lrrk2^+/+^*, S-*Lrrk2^-/-^*, SD-*Lrrk2^+/+^*, and SD-*Lrrk2^-/-^* groups. Original magnification 63 × (Vglut1) and with zoom = 2 (PSD 95); Scale bar = 20 μm. (**B**) Quantification of PSD95 volume per microglial soma, S-*Lrrk2^+/+^* group, N = 4 mice, n = 101 cells; SD-*Lrrk2^+/+^* group, N = 6 mice, n = 246 cells; S-*Lrrk2^-^*^/-^ group, N = 5 mice, n = 145 cells; SD-*Lrrk2^-^*^/-^ group, N = 3 mice, n = 36 cells. Data are plotted as mean ± SEM. ** *p* < 0.01, **** *p* < 0.0001, by two-way ANOVA with Tukey’s multiple comparisons test. (**C**) Quantification of Vglut1 volume per microglia soma, S-*Lrrk2^+/+^* group, N = 3 mice, n = 57 cells; SD-*Lrrk2^+/+^* group, N = 5 mice, n = 97 cells; S-*Lrrk2^-^*^/-^ group, N = 4 mice, n = 82 cells; SD-*Lrrk2^-^*^/-^ group, N = 3 mice, n = 58 cells. Data are plotted as mean ± SEM. ** *p* < 0.01, by *two-way* ANOVA with Tukey’s multiple comparisons test.

To verify whether SD interferes with microglia-mediated synaptic pruning, we examined the expression of immunofluorescence-labeled PSD95^+^ (post-synaptic density protein 95) and Vglut1^+^ (vesicular glutamate transporter (1) which were engulfed within Iba-1^+^ cells in the PFC. We quantified the internalization of PSD95^+^ and VGLUT1^+^ puncta within each of the microglial soma (Figure 4A). The results showed that compared with that in the S-*Lrrk2*^-/-^ mice, the volume of PSD95^+^ puncta within the microglia significantly increased in the SD-*Lrrk2*^-/-^ mice (*p* < 0.0001) (Figure 4B) After SD, the engulfment of PSD95^+^ puncta increased in the SD-*Lrrk2*^-/-^ group when in comparison with that of the SD-*Lrrk2*^+/+^ group (*p* < 0.001) (Figure 4B). The endocytosis rate of Vglut1 by the microglia significantly decreased in the S-*Lrrk2*^-/-^ mice when compared with that of the S-*Lrrk2*^+/+^ counterparts (*p* = 0.0033) (Figure 4C), but no statistical difference was observed between the SD-*Lrrk2*^+/+^ and SD-*Lrrk2*^-/-^ mice (Figure 4C).

### 3.5. LRRK2 Deficiency Induces the Abnormalities of Short-Term Memory

**Figure 5:**
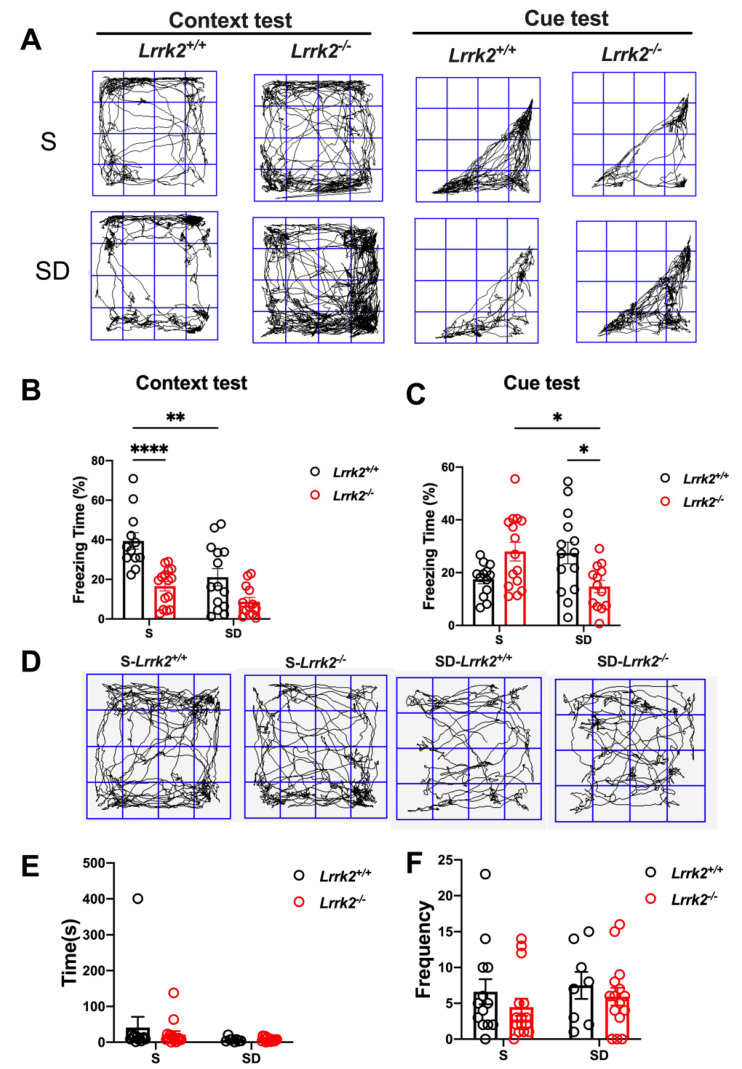
The abnormalities in short-term memory induced by LRRK2 deficiency. (**A**) Fear conditioning test, training track diagram; N = 13 and 14, respectively for 13-month-old S-*Lrrk2^+/+^* and SD-*Lrrk2^+/+^* mice; N = 15 and 12, respectively for 13-month-old S-*Lrrk2^-/-^* and SD-*Lrrk2^-/-^* mice. (**B**,**C**) Data are presented as mean ± SEM. * *p* < 0.05, ** *p* < 0.01, **** *p* < 0.0001, by Unpaired *t* test. (**D**) Representative traces of the open field test. (**E**,**F**) Data are presented as mean ± SEM and were analyzed by Tukey’s multiple comparisons test.

To determine whether SD affects the cognition of *Lrrk2^+/+^* and *Lrrk2*^-/-^ mice, a fear conditioning test was administered to assess the association between short-term memory and SD (Figure 5A). In the context test, the freezing time decreased in both the SD-*Lrrk2*^+/+^ (*p* = 0.0025) and S-*Lrrk2*^-/-^ (*p* < 0.001) groups when in comparison with that of the S-*Lrrk2^+/+^* mice (Figure 5B). In the cue test, no statistical difference in the cued recall memory was observed between the S-*Lrrk2*^-/-^ and the S-*Lrrk2^+/+^* groups (Figure 5C). Interestingly, the freezing time of the SD-*Lrrk2*^-/-^ group significantly decreased in the cue test when compared with that of the S-*Lrrk2*^-/-^ group (*p =* 0.0214) (Figure 5C). Notably, in the cue test, the freezing time of the SD-*Lrrk2*^-/-^ group significantly decreased when compared with that of the SD-*Lrrk2^+/+^* mice (*p* = 0.0336) (Figure 5C). These data evidence that the prefrontal cortex-amygdala-dependent learning and memory are impaired following the SD and the loss-of-function of LRRK2 especially impairs the cued recall memory and mediates distinct cognitive responses to SD.

To assess the impacts of SD on the activity and anxiety in mice, we examined the changes in anxiety behaviors (Figure 5D). Anxiety behavior was measured by the number of center entries and the time spent in the center of arena in the open field test. No significant difference was found among the groups (*p* > 0.05) (Figure 5E,F), indicating that the outcome of the fear conditioning test is not affected by the activity and anxiety level under the same conditions.

## 4. Discussion

No previous systematic study has probed into the changes in the sleep rhythm and cognition when LRRK2 deficiency is coupled with SD. After SD, LRRK2-deficient mice displayed lengthened NREM and shortened REM and worse cognitive function, particularly poorer recall memory cued by fear conditioning test. In addition, the increased microglial phagocytosis of postsynaptic material in the SD-Lrrk2^-/-^ mice was associated with the altered microglial activity that was involved in synaptic elimination, which is characterized by clear signs of inflammations. In this process, the pro-inflammatory cytokines (IL-1 and IL-6) were significantly elevated in the PFC of the SD-Lrrk2^-/-^ mice. Further analysis revealed that LRRK2 deficiency resulted in decreased dendritic spines, probably through microglial activation and synaptic endocytosis.

Sleep disorders are prevalent among the populace and may precede the onset of PD [5]. Evidence linking REM sleep to memory consolidation suggests that memory loss may be attributed to insufficient sleep syndrome and various parasomnias [25]. More specifically, REM sleep duration may be associated with either up-regulation or down-regulation of associated emotions encoded in the amygdala, which are strongly inhibited and regulated by the PFC [26,27]. REM sleep has previously been associated with the consolidation of spatial and emotional memory [12,28]. Sleep promotes the acquisition, integration, and consolidation of new information, and plays an essential role in modulating the overall synaptic connectivity, which intensifies during a waking phase [29]. Importantly, our results further demonstrate that after SD, LRRK2 knockout can alter the dendritic spinal integrity, synaptic density, and synapse-related signaling, and lead to synaptic dysfunction and cognitive impairment.

According to the current literature, synaptic loss seems to be a shared early feature of various neurodegenerative diseases [30]. Impaired glial clearance of damaged neurites has been found in sleep-deprived animals [31]. Recent studies have revealed that microglia play a crucial part in synaptic elimination, contributing to neurodegeneration-associated network dysfunction. Microglia-mediated synaptic loss also affects the excitatory and inhibitory neuron activity, leading to imbalanced synaptic transmission and subsequent synaptic dysfunction. In the current study, our results revealed that LRRK2 deficiency coupled with SD increased the number of microglia-mediated phagocytic events within the PFC. Previous studies document direct morphological microglial activation during a switch to an active ameboid state and evidence signs of microglial phagocytosis only after sleep deprivation, signifying that microglia are fully involved in severe sleep loss [32]. In our study, sleep deprivation in LRRK2-deficient mice altered their microglial activity, suggesting that LRRK2 may be crucial for sleep homeostasis. Of note, we found that LRRK2 deficiency did not increase the synaptic phagocytosis in the S-*Lrrk2^-/-^* mice, which was greatly promoted in *Lrrk2^-/-^* mice after SD.

Previous reports have demonstrated that chronic sleep disorders can trigger pathogenic processes that resemble the early onset of neurodegeneration, including dysfunction of endosome-autophagosome-lysosome pathway and microglia-mediated neuroinflammation [33]. Moreover, sleep loss has been reported to exert pro-inflammatory effects [32]. Recent studies show that IL-1β in *Lrrk2^-/-^* mice decreases after the S. Typhimurium infection [34]. Our data showed that IL-1β was not statistically different between the baseline sleep groups, but increased sharply (by about 10-fold) in the SD-*Lrrk2^-/-^* mice after SD. This alteration may be explained by the compensation from its homolog LRRK1 expressed in the brain or other pro-inflammation system [35]. Previous findings indicate that the depletion of microglia may decrease the nighttime wakefulness in mice by increasing transitions between NREM sleep and wakefulness [36]. Given that LRRK2 is highly expressed in microglia, it is likely that this gene contributes to the transition between wakefulness and NREM sleep via altered neuroinflammatory signaling.

Studies have reported that lysosomes can be recruited to the spinal head by the activity of a single dendritic spine. The blockage of the recruitment can reduce spinal density and altered lysosomal function may impact the maintenance of excitatory synapses [37]. LRRK2 regulates and controls lysosomal and endomembrane homeostasis [38,39]. LRRK2 and its substrate Rab GTPases are sequentially targeted onto stressed lysosomes [40]. However, it remains obscured whether LRRK2 and its substrate Rab GTPases are necessary for sleep deprivation-mediated microglial phagocytosis. Further studies are awaited to probe into the link of the LRRK2-related lysosome changes to sleep loss.

However, due to the limitation of experimental conditions, we could not record and understand the electrophysiological changes of excitatory or inhibitory neurons under the condition of LRRK2 deficiency coupled with sleep deprivation, which is also the direction of our follow-up research. More experiments are also needed to uncover the projections of PFC to other regions with high levels of LRRK2 expression, and their specific interactions with the temporally precise record of the memory-associated rhythm during REM and NREM sleep.

## 5. Conclusions

Our findings provide insights into the physiological role of LRRK2-mediated pathways in regulating microglial synaptic pruning with respect to modulating SD-related cognitive disorders. Current evidence is also supportive of the hypothesis that LRRK2 activity is essential for REM/NREM sleep stability and SD-related cognition, which is mediated by microglial synaptic refinement in neurodegenerative diseases.

## Data Availability

The data used for this study, though not available in a public repository, will be made available from the corresponding author upon reasonable request.

## Abbreviations

CD68, Cluster of differentiation 68; DLB, dementia with Lewy bodies; EEG, electroencephalogram; EMG, electromyogram; IL-1, interleukin 1; IL-4, interleukin 4; IL-6, interleukin 6; iPD, idiopathic Parkinson’s disease; iRBD, isolated rapid-eye-movement sleep behavior disorder; IRBD, idiopathic REM sleep behavior disorder; LRRK2, leucine rich repeat kinase 2; NREM, nonrapid eye movement; PD, Parkinson’s disease; PFC, prefrontal cortex; PSD95, post-synaptic density protein 95; REM, rapid eye movement; S, normal sleep; SD, sleep deprivation; VGLUT1, vesicular glutamate transporter.
